# Fibronectin glomerulopathy in a kidney allograft biopsy

**DOI:** 10.1186/s12882-023-03403-y

**Published:** 2023-12-05

**Authors:** Nathaniel Klair, Salman B. Mahmood, Rasha El-Rifai, Cynthia C. Nast, Lihong Bu, Adam Bregman

**Affiliations:** 1 Department of Internal Medicine, University of Minnesota Medical Center, Minneapolis, MN USA; 2 Division of Nephrology and Hypertension, Department of Medicine, University of Minnesota Medical Center, 717 Delaware St SE, Minneapolis, MN 55414 USA; 3 Department of Pathology and Laboratory Medicine, Cedars-Sinai Medical Center, Los Angeles, CA USA; 4 Department of Laboratory Medicine and Pathology, Mayo Clinic, Rochester, MN USA

**Keywords:** Fibronectin glomerulopathy, FN1 mutation, Kidney biopsy

## Abstract

**Background:**

Fibronectin glomerulopathy is a rare genetic nephropathy with only a few cases of post-transplant recurrence being reported previously. We highlight a case that was initially misdiagnosed and emphasize the importance of full immunofluorescence and electron microscopy evaluation in allograft biopsies.

**Case presentation:**

A 36-year-old male with a history of end-stage kidney disease secondary to biopsy-proven type 1 membranoproliferative glomerulonephritis (MPGN) status-post living unrelated donor kidney transplant 12 years prior, presented with increasing creatinine and proteinuria. Biopsy was performed and was consistent with fibronectin glomerulopathy. Subsequent genetic testing revealed an FN1 mutation, the primary gene associated with this condition.

**Conclusions:**

Full histologic evaluation of the allograft biopsy corrected the diagnosis and additionally suggested that the patient's mother, who had expired in her 30s and had received a diagnosis of type 1 MPGN on autopsy, likely also had fibronectin glomerulopathy, enabling appropriate genetic counseling for the family.

## Background

Fibronectin glomerulopathy (FG) is a rare genetic nephropathy characterized by deposition of fibronectin in the glomeruli leading to proteinuria, hypertension, hematuria, and progressive chronic kidney disease (CKD) [1]. Most reported cases are familial, autosomal dominant, and associated with a mutation in the FN1 gene [2, 3], which encodes fibronectin, an extracellular matrix protein. Several different mutations in FN1 have been reported to lead to FG. The mutations are thought to interfere with beta sheet formation which then leads to fibronectin deposition [4].

The diagnosis of FG depends on kidney biopsy and can be mislabeled as membranoproliferative glomerulonephritis (MPGN) [5, 6], which describes a pattern of injury but not the underlying pathogenesis. As patients with FG often progress to end stage kidney disease (ESKD), an erroneous diagnosis can have important implications for care post-transplant. We herein present such a case, where the correct diagnosis of FG was ultimately determined several years after kidney transplant.

## Case report

A 36-year-old male presented with progressive CKD after kidney transplant.

His history began at age five when he was incidentally found to have proteinuria during a workup for developmental delay. He underwent kidney biopsy and was diagnosed with primary type 1 MPGN, though it was noted at the time that he had normal complement levels. Family history was notable for his mother also being diagnosed with MPGN type 1. He developed progressive CKD, and ultimately ESKD necessitating initiation of hemodialysis at age 17. At age 23 he received a living unrelated donor kidney transplant.

**Fig. 1:**
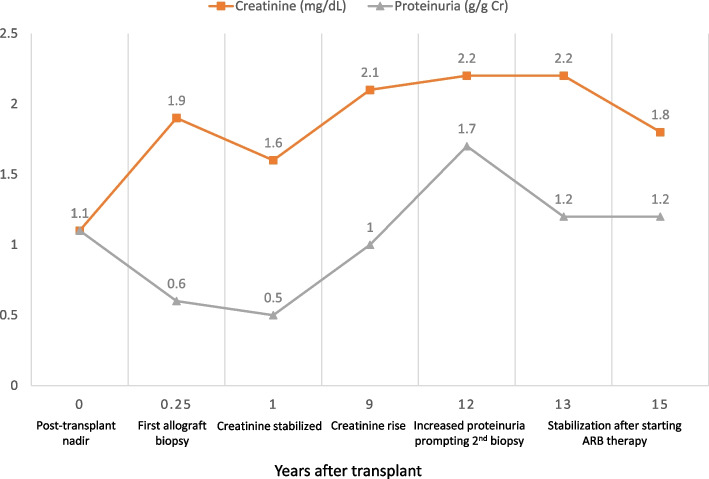
Creatine and proteinuria trends post-transplant

His post-transplant creatinine nadir was 1.1 mg/dL; however, in the two months following transplant, his creatinine progressively increased, stabilizing in the 1.8–2.2 mg/dL range (see Fig. 1 for creatinine and proteinuria trends). Workup at that time, including kidney biopsy, was unrevealing—notably there were no signs of rejection or glomerular disease, and specifically there were no deposits noted on immunofluorescence (IF) staining or electron microscopy (EM).

His immunosuppression followed a standard protocol consisting of cyclosporine (Neoral®) and mycophenolate. His maintenance regimen was cyclosporine (goal trough 75–100 mcg/L) and mycophenolic acid 540 mg twice daily. He was on trimethoprim-sulfamethoxazole for Pneumocystis jirovecii pneumonia prophylaxis. Post transplant hypertension was managed with a calcium channel blocker and a beta blocker due to previous issues with hyperkalemia on an ACE inhibitor (ACEi). His medical history is also notable for hypothyroidism, intellectual disability, and secondary hyperparathyroidism.

**Fig. 2:**
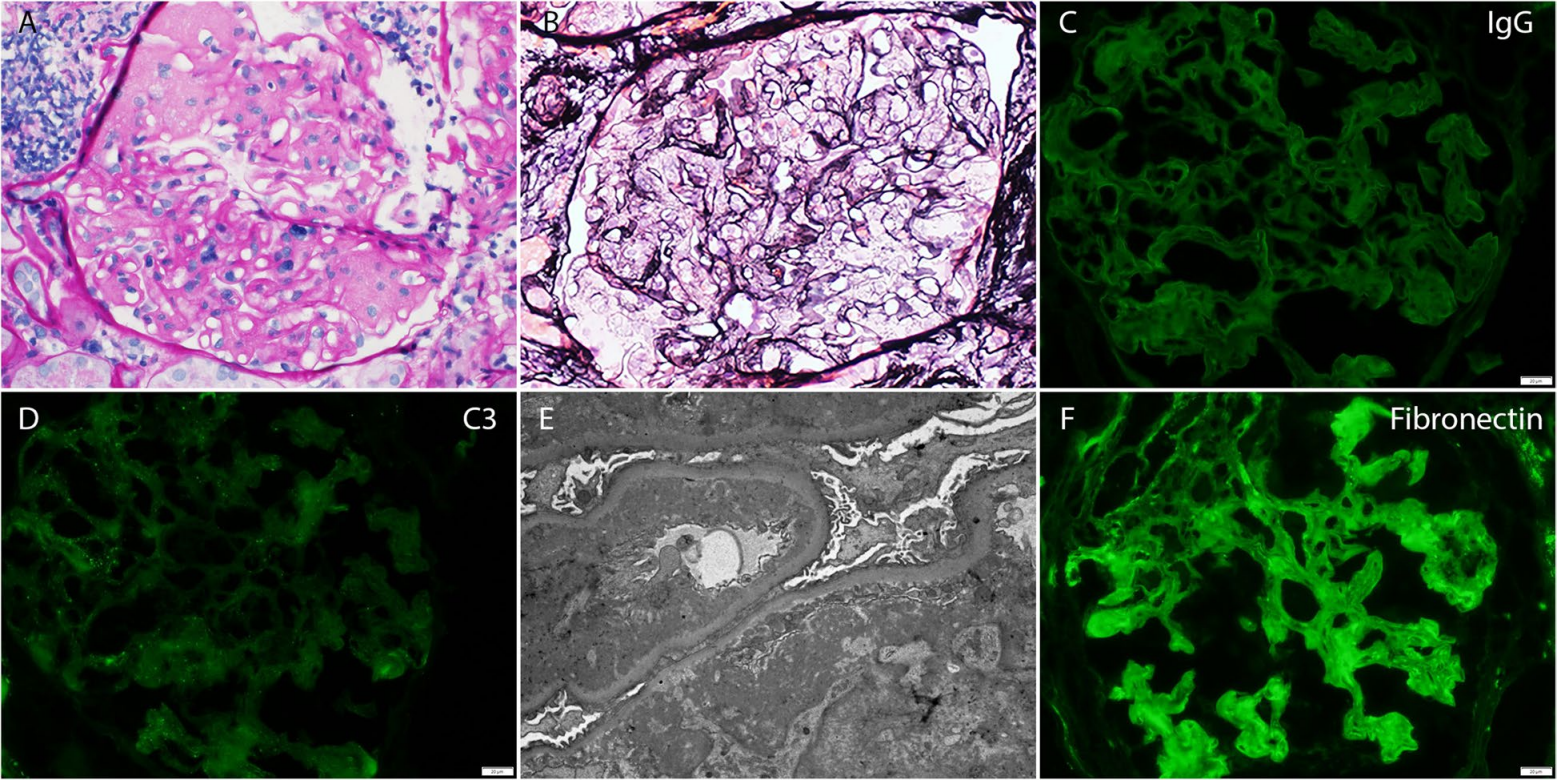
Allograft kidney biopsy findings of fibronectin nephropathy. Light microscopy shows lobular accentuation with marked mesangial expansion, mild mesangial hypercellularity, and variable expansion of glomerular basement membranes by periodic acid-Schiff-positive (**A**) and silver-negative material (**B**). Congo red stain is negative (not shown). No necrosis, crescents, segmental sclerosis, glomerular basement membrane spikes or double contours, pseudothrombi or vasculitis are present. By routine immunofluorescence, there is no significant glomerular or extra-glomerular staining for IgG (**C**), IgA, IgM, C1q, C3 (**D**), fibrinogen, kappa, or lambda. The negative immunoglobulin and complement staining are confirmed by immunofluorescence performed on paraffin tissue sections after pronase digestion. Ultrastructurally, there are massive mesangial and subendothelial electron dense deposits without evident substructure (**E**). Fibronectin immunofluorescence stain is positive in deposits (**F**). Original magnification, × 200 for A and B, × 400 for **C**, **D**, and **F**

At 12 years post-transplant, proteinuria was noted to have increased from 1.0 g/g Cr to 1.7 g/g Cr and the serum Cr simultaneously increased to 2.2 mg/dL prompting repeat kidney allograft biopsy (Fig. 2).

Light microscopy showed a membranoproliferative pattern of injury with lobular accentuation with marked mesangial expansion and variable thickening of glomerular basement membranes by silver-negative and periodic acid-Schiff-positive material. There was no significant glomerular or extraglomerular staining by routine and pronase IF staining. However, EM showed massive electron dense deposits in mesangial and subendothelial regions with a focal vague fibrillar appearance. Subsequently, IF staining for fibronectin was performed and was positive in the deposits (Fig. 2). These findings were consistent with FG. Genetic testing was performed using the commercially available, CAP acrredited Renasight CKD Gene Panel which tests for 385 gene associated with CKD. The testingrevealed an FN1 mutation, the primary gene associated with FG. This consisted of an A to G substitution leading to a missense Tyr to Cys change at codon 973 in exon 19 of the FN1 gene, (NM_212482.2:c.2918A > G(p.Tyr973Cys), Pathogenic mutation) that has been previously described [1].

Recommended treatment of FG is general CKD management. There is no role for immunosuppression. Therefore with the increased proteinuria, the patient was successfully initiated on an angiotensin receptor blocker (ARB) despite prior hyperkalemia on an ACEi. Fortunately, the proteinuria and creatinine returned to previous baselines (Fig. 1). The patient was additionally referred for genetic counseling. Table 1 is a timeline of major events in this case.

**Table 1:** Chronological outline of the case events

Age	Event
5y	Proteinuria, biopsy with Type 1 MPGN
17y	ESRD, initiated on hemodialysis
23y8m	Living non-related kidney transplant
23y8m	Post-transplant Cr nadir of 1.1 mg/dL on post-op day 5
23y10m	Negative allograft biopsy (no deposits on EM)
36y0m	Creatinine increased to 2.2 mg/dL and Proteinuria to 1.7 g/g Cr
36y0m	Recurrent FG on allograft biopsy
36y1m	Genetic testing confirmed FG
36y2m	Started on ARB
38y8m	Most recent creatinine 1.8 mg/dL and proteinuria 1.2 g/g Cr

## Discussion

The characteristic biopsy findings combined with the FN1 gene mutation confirmed the diagnosis of FG. FG can have a variety of histologic patterns, one of which is MPGN on light microscopy, and as a result may be initially mislabeled as MPGN as occurred in this case [7–9]. Of note, the FN1 mutation exhibits incomplete penetrance; nevertheless, it is likely the patient's mother, who had been diagnosed with MPGN, almost certainly also had FG [3]. Fibronectin has both a soluble, plasma-derived form and an insoluble, cellular form. In FG, the soluble form deposits in the glomerulus causing kidney injury. This was determined by experiments using antibodies specific to the two forms [8]. FG can recur after transplant because soluble fibronectin still circulates and deposits in the allograft despite the absence of an FN1 genetic mutation in the donor [8]. An MPGN pattern of glomerular injury is almost always secondary to an underlying systemic disease process, related to immune complex deposition, complement dysregulation, or a non-immune complex non-complement mechanism of disease. Patients with an MPGN pattern of injury have been reported to have genetic risks factors [10] and there are reported cases of familial disease with histological findings of MPGN, particularly associated with complement dysregulation [11]. However, most cases with an MPGN pattern of injury are not directly inherited. In contrast, the majority of described cases of FG are hereditary, with a few reports of sporadic cases [12]. Additionally, therapeutic considerations for an MPGN lesion depend directly on the underlying etiology and include a number of immunosuppressive medications [10], which would not be expected to have efficacy in FG, for which there are no specific treatments.

MPGN is reported to recur 20–40% of the time after transplant, although these studies were done before the reclassification of MPGN by immunofluorescence findings and pathogenesis [13, 14].

There is much less data on the recurrence of FG. To our knowledge, only five such cases have been reported previously (Table 2) [1, 2, 8, 15, 16]. In these cases, proteinuria was detected within months to a few years post-transplant, consistent with our findings. In two of these cases the diagnosis of FG was made on the allograft biopsy. One case was misdiagnosed as mesangial proliferative glomerulonephritis on native biopsy [16] and in the other no native biopsy was performed [15]. Further understanding the risk of recurrence after transplant is important for predicting the future clinical course.

**Table 2:** Reports of recurrent FG in transplanted kidneys

Report	Initial diagnosis	Age at initial diagnosis / Sex	Age at ESKD	Recurrence description	Genetic mutations identified	Allograft biopsy EM Histologic
Castelletti (2008) [1]	FG by native biopsy	18 M	32	Recurrence in allograft biopsy 3 years post- transplant	NM_212482.4:c.5775G > C (p.Trp1925Cys) NM_212482.3:c.5921T > C(p.Leu1974Pro) NM_212482.2:c.2918A > G(p.Tyr973Cys)	"the deposits were mainly granular"
Gemperle (1996) [2]	FG by native biopsy	31 M	45	Proteinuria at 7 months post-transplant, allograft biopsy at 23 months post-transplant confirmed recurrence	No genetic mutation identified	"giant subendothelial fibrillary deposits in the allograft"
Strom (1995) [8]	FG by native biopsy	31 M	N/A	Recurrence in allograft biopsy	No genetic testing performed	"relapse of deposits"
Otsuka (2012) [15]	Unknown (no native biopsy performed)	Teenage years F	49	Recurrence in allograft biopsy 12 months post-transplant	No genetic testing performed	"electron-dense deposits with microtubular structure measuring 12–14 nm in width"
Wei (2022) [16]	Mesangial proliferative glomerulonephritis with membranous-like changes by native biopsy	47 F	60	Proteinuria at 27 months post-transplant, allograft biopsy then led to FG diagnosis	NM_212482.3:c.5921T > C(p.Leu1974Pro)	"obscure granular or short fibrillar appearance with a much higher density"
This case	Type 1 MPGN by native biopsy	5 M	17	Proteinuria noted within months post-transplant, allograft biopsy at 12 years post-transplant led to FG diagnosis	NM_212482.2:c.2918A > G(p.Tyr973Cys)	"massive mesangial and subendothelial electron dense deposit"

In summary, if IF and EM had not been performed on the transplant kidney biopsy, an erroneous diagnosis of recurrent MPGN would have persisted. We emphasize the importance of full histologic evaluation in allograft biopsies for recognition of potentially recurrent glomerular diseases that may have been missed previously on native kidney biopsies or in patients in whom such biopsies were not performed. This may have significant implications for treatment, prognosis, knowing the risk of disease recurrence in future kidney transplants, and providing family members with important information if a genetic disease is identified.

## Data Availability

All data generated or analyzed during this study are included in this published article.

## Abbreviations

MPGN: Membranoproliferative glomerulonephritis
FG: Fibronectin glomerulopathy
CKD: Chronic kidney disease
ESKD: End stage kidney disease
IF: Immunofluorescence
EM: Electron microscopy
ACEi: ACE inhibitor
ARB: Angiotensin receptor blocker
